# Quality of Diabetes Care in Germany Improved from 2000 to 2007 to 2014, but Improvements Diminished since 2007. Evidence from the Population-Based KORA Studies

**DOI:** 10.1371/journal.pone.0164704

**Published:** 2016-10-17

**Authors:** Michael Laxy, Gabriella Knoll, Michaela Schunk, Christa Meisinger, Cornelia Huth, Rolf Holle

**Affiliations:** 1 Institute of Health Economics and Health Care Management, Helmholtz Zentrum München, Neuherberg, Germany; 2 German Center for Diabetes Research (DZD), München-Neuherberg, Germany; 3 Institute for Medical Informatics, Biometrics and Epidemiology, Ludwig-Maximilians- Universität München, Munich, Germany; 4 Institute of Epidemiology II, Helmholtz Zentrum München, Neuherberg, Germany; Virginia Commonwealth University, UNITED STATES

## Abstract

**Objective:**

Little is known about the development of the quality of diabetes care in Germany. The aim of this study is to analyze time trends in patient self-management, physician-delivered care, medication, risk factor control, complications and quality of life from 2000 to 2014.

**Methods:**

Analyses are based on data from individuals with type 2 diabetes of the population-based KORA S4 (1999–2001, n = 150), F4 (2006–2008, n = 203), FF4 (2013/14, n = 212) cohort study. Information on patient self-management, physician-delivered care, medication, risk factor control and quality of life were assessed in standardized questionnaires and examinations. The 10-year coronary heart disease (CHD) risk was calculated using the UKPDS risk engine. Time trends were analyzed using multivariable linear and logistic regression models adjusted for age, sex, education, diabetes duration, and history of cardiovascular disease.

**Results:**

From 2000 to 2014 the proportion of participants with type 2 diabetes receiving oral antidiabetic/cardio-protective medication and of those reaching treatment goals for glycemic control (HbA1c<7%, 60% to 71%, p = 0.09), blood pressure (<140/80 mmHg, 25% to 69%, p<0.001) and LDL cholesterol (<2.6 mmol/l, 13% to 27%, p<0.001) increased significantly. However, improvements were generally smaller from 2007 to 2014 than from 2000 to 2007. Modeled 10-year CHD risk decreased from 30% in 2000 to 24% in 2007 to 19% in 2014 (p<0.01). From 2007 to 2014, the prevalence of microvascular complications decreased and quality of life increased, but no improvements were observed for the majority of indicators of self-management.

**Conclusion:**

Despite improvements, medication and risk factor control has remained suboptimal. The flattening of improvements and deteriorations in quality of (self-) care since 2007 indicate that more effort is needed to improve quality of care and patient self-management. Due to selection or lead time bias an overestimation of quality of care improvements cannot be ruled out.

## Background

Diabetes mellitus is a major public health problem and one of the biggest challenges for healthcare systems all over the world [1]. The prevalence of known type 2 diabetes in the German adult population in 2010 was 7–8% and in addition it is to consider that a high number of patients are not yet diagnosed with the disease [2, 3]. Due to an aging population and unhealthy lifestyle the prevalence of type 2 diabetes is expected to increase steadily over the next decades.

The medical consequences of diabetes and its complications impose a huge burden on both the affected individuals and the society as a whole. In Germany, patients with diabetes have around 1.8 times higher health care costs than people without diabetes resulting in annual excess costs of around 21 billion €. Around 75% of these excess costs are attributable to the treatment of diabetic complications [4]. Besides causing financial pressure on the health care system, diabetes affects the patients’ health-related quality of life (HRQL) and decreases their life expectancy. Particularly, patients with micro- and macrovascular complications suffer from a disproportionate HRQL and mortality burden [5–7].

Large randomized controlled trials in patients with type 2 diabetes have shown that the control of blood glucose, blood pressure and lipids are effective strategies to reduce the risk for diabetic complications [8–13]. These strategies are furthermore considered to be highly cost-effective [14–17]. To achieve good risk factor control, continuing physician-delivered medical care, including monitoring of risk factors, patient education and appropriate medication, as well as active patient self-management are needed. Large efforts have been made to improve the quality of diabetes care in Germany. For example in 2002, structured disease management programs (DMPs) for type 2 diabetes were rolled out nationwide within the system of statutory health insurances in order to reinforce guideline-based care [18–20].

However, despite these efforts, there is little evidence on the development of the quality of diabetes care in Germany. German health insurance claims data only comprise information about diagnoses and procedures but do not contain information on clinical variables, such as HbA1c, or information on lifestyle habits or patient self-management behavior. Evidence on quality of care is therefore largely based on data from population-based studies. These studies showed that medication and control of blood glucose and blood pressure increased in the time between 1997 and 2011, but that quality of care remained suboptimal [21–24]. However, few studies reported data for the last 5 years and therefore very little is known about recent trends. Furthermore, although patient self-management is known to be an important pillar in the disease management process, reliable data on the development of patient self-management is scarce [25].

The goal of this study was to evaluate time trends of patient self-management, physician-delivered care, medication, risk factor control and quality of life from 2000 to 2014 using population-based survey data.

## Research Design and Methods

### Study sample

**Fig 1:**
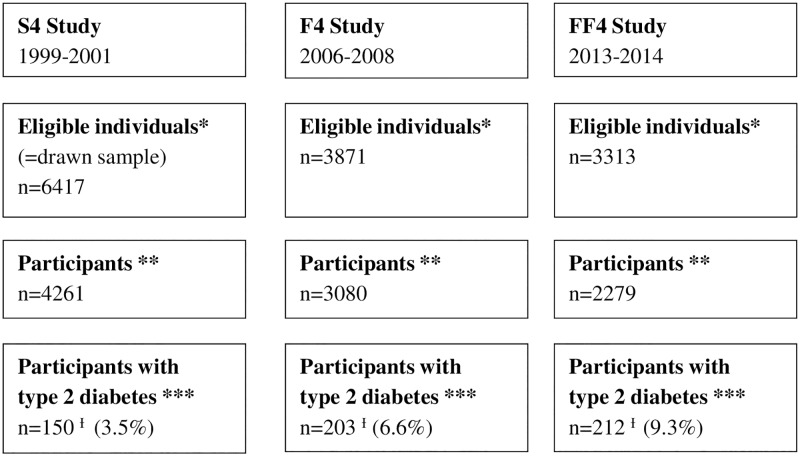
Study design. * eligible individuals; ** Individuals who actually participated in the study; *** Individuals with a validated diagnosis of type 2 diabetes and who reported to have diabetes; Ɨ Due to new incident cases and dropout of prevalent cases the altogether 565 observations with type 2 diabetes stem from 375 different individuals: 64 only S4, 50 only F4, 106 only FF4, 48 S4 and F4, 69 F4 and FF4, 3 S4 and FF4, 35 S4, F4 and FF4.

The analyses of this study are based on data from the KORA (Cooperative Health Research in the Region of Augsburg) S4/F4/FF4 cohort study. The KORA S4 study was conducted in the years 1999–2001. From 6417 eligible community dwelling individuals aged 25–74 who were randomly selected from population registries in the city of Augsburg and two surrounding counties 4261 participated in the baseline study (S4). In the years 2006–2008, and 2013/2014 S4-participants were re-invited and 3080 out of 3871 eligible individuals and 2279 out of 3313 eligible individuals took part in a 1^st^ follow-up (F4) and 2^nd^ follow-up examination (FF4), respectively. The study design is illustrated in Fig 1. All three studies were approved by the Ethics Committee of the Bavarian Medical Association and all study participants provided written informed consent. Study design, sampling method and data collection have been described in detail elsewhere [26].

### Participants with type 2 diabetes

Participants with a validated diagnosis of type 2 diabetes (validation from responsible physician) and who reported at the same time to have diabetes, i.e. participants who were aware of their disease, were included in the study. Following this definition 150 participants out of 4261 (3.5%) from the baseline study (S4: 1999–2001), 203 participants out of 3080 participants (6.6%) from the 1^st^ follow-up study (F4: 2006–2008), and 212 participants out of 2279 (9.3%) from the 2^nd^ follow-up study (FF4: 2013/14) were included.

Due to new incident cases and dropout of prevalent cases the 565 observations stem from 375 different participants with prevalent type 2 diabetes. With this, the data structure is based on a mixture of a panel and a repeated cross-sectional study design. The participant flow is described in detail in Fig 1.

### Quality of Care Measures

#### Patient self-management

During their visit to the study center participants with diabetes were asked to fill out a questionnaire regarding their self-management behavior. Participants were asked if they monitor and check their blood glucose, blood pressure, weight and feet at least once a week, if they currently smoke, are physically active at least 2 hours per week, if they currently keep a diabetes diary or have ever attended a diabetes education class.

#### Physician-delivered care

In the self-administered questionnaire participants were also asked whether a physician has at least once over the last 12 months measured their HbA1c, blood pressure, cholesterol level and protein in urine, examined their eyes or feet or advised them on the lifestyle topics diet and physical activity. The wording of questions on patient self-management and physician-delivered care was the same in all three studies; however, some questions and items were added in F4 and FF4.

#### Medication

Participants were asked to bring the original packaging of the pharmaceutical products taken during the last 7 days prior to the examination. Based on this information, Anatomical Therapeutic Chemical Classification System codes were assigned to define the use of oral antidiabetic medication, insulin, blood pressure lowering medication, lipid lowering medication and platelet inhibiting medication.

#### Clinical outcome measures

Participants were asked to come in fasting on the morning of examination. Physical examinations included anthropometric measurements, and blood draw. Body mass index (BMI), diastolic and systolic blood pressure, HbA1c, total cholesterol, LDL cholesterol, HDL cholesterol and triglyceride values were assessed. A detailed overview on used laboratory methods is provided in S1 Table. Subsequently, all clinical outcome measures were dichotomized based on German [27] and international guidelines [28] of the year 2014.

#### Ten-year modeled risk for stroke and CHD

For participants with type 2 diabetes and without a history of CVD events (no previous myocardial infarction or stroke) the ten-year coronary heart disease (CHD) and stroke risks were calculated using the UK Prospective Diabetes Study risk engine (UKPDS Risk Engine, Oxford University Innovation Ltd 2001). The UKPDS risk engine is a risk-assessment tool that estimates the absolute risk of fatal or non-fatal CHD and stroke within a defined time frame in individuals with type 2 diabetes not known to have heart disease. The risk engine is based on the factors age, sex, ethnicity, smoking status, diabetes duration, HbA1c, systolic blood pressure, total cholesterol, HDL cholesterol, and atrial fibrillation. As information on atrial fibrillation was not available for all 3 studies, all values were set to zero (no atrial fibrillation).

#### Microvascular complications and health-related quality of life

Participants were asked if a physician has ever made a diagnosis of ‘retinopathy’, ‘protein in urine’ (proteinuria), or ‘neuropathy in their legs’. HRQL was assessed with the SF-12 questionnaire. The SF-12 contains 6 physical- and 6 mental health-related questions across 8 empirically distinct health domains and can be summarized in a Physical and Mental Component Summary (PCS and MCS).

### Statistical Analyses

Analyses are based on 565 observations of 375 individuals with type 2 diabetes. We applied General Estimation Equation (GEE) models with an autoregressive covariance structure to account for the partially repeated measurement structure of the data using SAS 9.4 (SAS Institute, Cary, North Carolina, USA). Time (ordinal variable defined as S4, F4 and FF4) was introduced as a fixed effect into the model. A logit link with a binary distribution (logistic regression) was used for dichotomized outcomes and an identity link with a Gaussian distribution (linear regression) for continuous variables. The LSMESTIMATE statement was used to estimate differences in adjusted means for continuous outcomes and adjusted odds ratios for binary outcomes for the comparisons 2007 vs. 2000, 2014 vs. 2007, and 2014 vs. 2000. To avoid shrinkage of data, missing information on duration of diabetes was imputed using a mean-based imputation method. All analyses were adjusted for age, age^2^, sex, education, diabetes duration and history of CVD (stroke, myocardial infarction)–information that was routinely collected in standardized interviews and questionnaires. Because some participants of the S4 study came to the examination non-fasting, regression models for lipids and modeled CHD/stroke risk were additionally adjusted for the fasting status of participants.

## Results

### Characteristics of the Study Population

Characteristics of the study population are described in Table 1. The mean duration of diabetes was between 8.6 years and 10.2 years in all 3 studies. However, with a mean age of approximately 62 years, the age in the S4 study was about 6 and 8 years lower than in the F4 and FF4 study, respectively.

**Table 1:** Characteristics of participants with type 2 diabetes.

	S4 study (1999–2001)	F4 study (2006–2008)	FF4 study (2013–2014)
n	150	203	212
Sex (men), n (%)	82 (54.7)	115 (56.7)	123 (58.0)
Age (years), mean (std)	61.9 (9.2)	67.6 (9.9)	69.7 (9.9)
Education, n (%)			
primary	128 (85.3)	154 (75.9)	144 (67.9)
secondary	10 (6.7)	23 (11.3)	33 (15.6)
tertiary	12 (8.0)	26 (12.8)	35 (16.5)
Diabetes duration (years), mean (std)	8.6 (7.4)	9.1 (8.1)	10.2 (7.5)
History of CVD, n (%)	19 (12.7)	31 (15.3)	41 (19.3)

CVD: myocardial infarction or stroke; std: standard deviation

### Patient self-management

**Fig 2:**
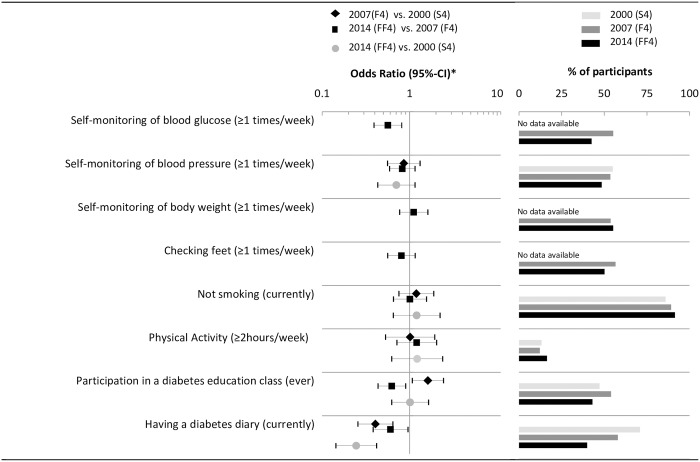
Time trends of patient self-management. * Logistic regression model adjusted for sex, age, age^2^, education, diabetes duration, and history of CVD.

Results on patient self-management are summarized in Fig 2. No significant trends on smoking, physical activity and self-monitoring of weight, blood pressure and feet or participation in a diabetes education class were observed from 2000 to 2014. However, the proportion of participants keeping a diabetes diary decreased from 71% (2000) to 58% (2007) to 40% (2014) (OR_2014 vs. 2000_ = 0.25 [95% CI: 0.14, 0.52]). Furthermore, the proportion of participants with diabetes who regularly self-monitor their blood glucose (OR_2014 vs. 2007_ = 0.56 [0.39, 0.81]) decreased significantly from 2007 to 2014.

### Physician-delivered care

**Fig 3:**
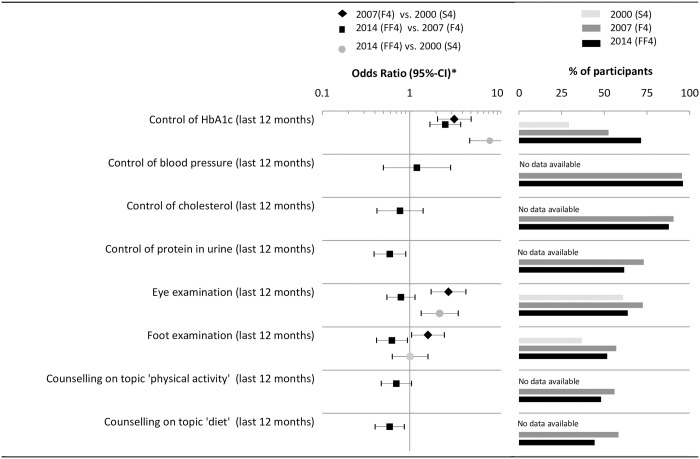
Time trends of physician delivered care. * Logistic regression model adjusted for sex, age, age^2^, education, diabetes duration, and history of CVD.

Fig 3 illustrates the results on physician-delivered care and medication. There was a significant increase in the reported annual HbA1c checks from 2000 (29%) to 2007 (53%) to 2014 (72%). The proportion of participants with type 2 diabetes reporting an eye and foot examination increased between 2000 and 2007 but decreased between 2007 and 2014. Furthermore, compared to 2007 in 2014 participants with diabetes were less likely to receive a check for proteinuria (OR_2014 vs. 2007_ = 0.59 [0.39, 0.90]) and counseling on diet (OR_2014 vs. 2007_ = 0.59 [0.40, 0.86]).

### Medication

**Fig 4:**
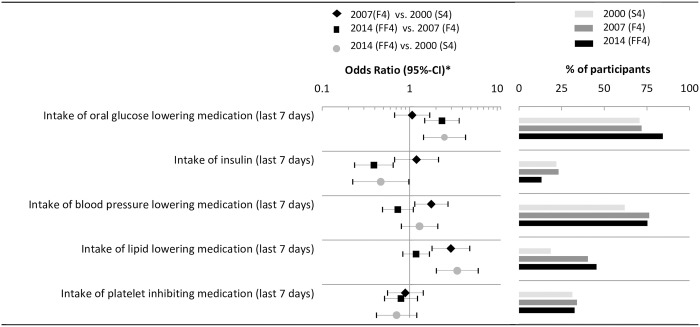
Time trends of medication use. * Logistic regression model adjusted for sex, age, age^2^, education, diabetes duration, and history of CVD.

Results on medication and risk factor control are depicted in Fig 4. Generally, the proportion of participants with diabetes receiving oral antidiabetic medication (OR_2014 vs. 2000_ = 2.50 [95%-CI: 1.44, 4.34]), blood pressure lowering medication and lipid lowering medication (OR_2014 vs. 2000_ = 3.50 [95% CI: 2.02, 6.06]) increased and the use of insulin significantly decreased from 2000 to 2014 (OR_2014 vs. 2000_ = 0.47 [95% CI: 0.22, 0.98]). Noteworthy, the increase in the proportion of patients receiving blood pressure lowering and lipid lowering medication is attributable to a large increase between 2000 and 2007 –little or no increases were observed between 2007 and 2014.

### Reaching treatment targets

**Fig 5:**
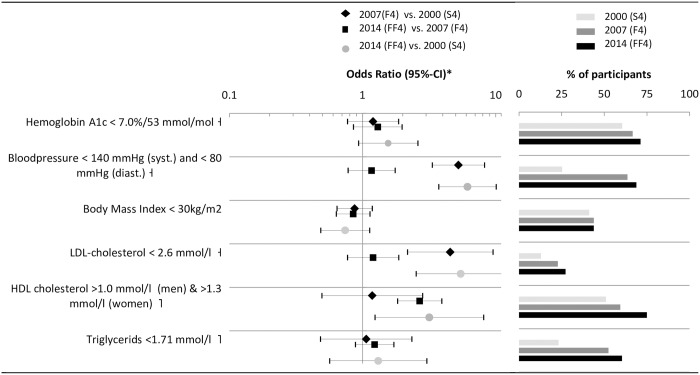
Time trends of reaching treatment targets. * Logistic regression model adjusted for sex, age, age^2^, education, diabetes duration, and history of CVD; ⊣ based on German guidelines 2014 [29]; ⌉ based on International guidelines 2014 [28].

The proportion of participants with diabetes reaching targeted goals for glycemic control (HbA1c< 7%, OR_2014 vs. 2000_ = 1.56 [0.93, 2.6]), blood pressure control (OR_2014 vs. 2000_ = 6.14 [95%-CI: 3.73, 10.09], LDL cholesterol (OR_2014 vs. 2000_ = 5.47 [95%-CI: 2.52, 11.84], and HDL cholesterol (OR_2014 vs. 2000_ = 3.17 [95%-CI: 1.24, 8.13], increased substantially from 2000 to 2014 (Fig 5). Except for HDL cholesterol, improvements from 2007 to 2014 were substantially smaller than those from 2000 to 2007.

### Modeled 10 year CHD and stroke risk

Table 2 shows that mean HbA1c fell from 7.1% to 6.9% to 6.8%, systolic blood pressure from 145 mmHg to 132 mmHg to 127 mmHg, total cholesterol from 6.11 mmol/l to 5.14 mmol/l to 5.10 mmol/l and that HDL changed from 1.31 mmol/l to 1.25 mmol/l to 1.50 mmol/l from the year 2000 to 2007 to 2014. As a consequence, in participants with diabetes and without CVD history the modeled 10-year CHD and stroke risks, which are basically functions of these four parameters fell in the same time horizon from 30% to 24% to 19% (p<0.01) and from 19% to 16% to 14% (p<0.01), respectively.

**Table 2:** Time trends for intermediate outcomes and 10 year modeled cardiovascular risk.

	S4 study	F4 study	FF4 study	Association		
	1999–2001	2006–2008	2013/2014			
	Adjusted mean	Adjusted mean	Adjusted mean	Comparison	Adjusted difference	[95%- CI]
**HbA1c, blood pressure, BMI, and lipids** Ɨ						
HbA1c (%)	7.09	6.87	6.75	2007 vs. 2000	-0.23	[-0.49, 0.04]
				2014 vs. 2007	-0.12	[-0.31, 0.06]
				2014 vs. 2000	-0.35	[-0.63, -0.06]
Systolic blood pressure (mmHg)	145.3	131.6	126.8	2007 vs. 2000	-13.64	[-17.71, -9.56]
				2014 vs. 2007	-4.87	[-8.18, -1.57]
				2014 vs. 2000	-18.51	[-23.05, -13.97]
Diastolic blood pressure (mmHg)	82.48	73.63	72.8	2007 vs. 2000	-8.85	[-10.91, -6.80]
				2014 vs. 2007	-0.85	[-2.61, 0.90]
				2014 vs. 2000	-9.71	[-12.09, -7.32]
BMI (kg/m2)	31.67	31.57	31.5	2007 vs. 2000	-0.10	[-1.23, 1.02]
				2014 vs. 2007	-0.09	[-1.09, 0.92]
				2014 vs. 2000	-0.19	[-1.35, 0.97]
Total Cholesterol (mmol/l)	6.11	5.14	5.09	2007 vs. 2000	-0.97	[-1.31, -0.63]
				2014 vs. 2007	-0.05	[-0.22, 0.12]
				2014 vs. 2000	-1.02	[-1.38, -0.66]
LDL-cholesterol (mmol/l)	3.81	3.16	3.09	2007 vs. 2000	-0.65	[-0.93, -0.37]
				2014 vs. 2007	-0.08	[-0.23, 0.07]
				2014 vs. 2000	-0.73	[-1.03, -0.42]
HDL-cholesterol (mmol/l)	1.31	1.24	1.45	2007 vs. 2000	-0.07	[-0.19, 0.05]
				2014 vs. 2007	0.21	[0.16, 0.26]
				2014 vs. 2000	0.14	[0.00, 0.27]
Triglycerides (mmol/l)	2.27	2.01	1.92	2007 vs. 2000	-0.26	[-0.53, 0.01]
				2014 vs. 2007	-0.08	[-0.27, 0.10]
				2014 vs. 2000	-0.34	[-0.65, -0.04]
**Modeled 10-year CHD and stroke risk using the UKPDS risk engine** #						
UKPDS 10-year CHD risk	0.300	0.243	0.193	2007 vs. 2000	-0.057	[-0.098, -0.017]
				2014 vs. 2007	-0.050	[-0.067, -0.032]
				2014 vs. 2000	-0.107	[-0.150, -0.064]
UKPDS 10-year stroke risk	0.192	0.160	0.140	2007 vs. 2000	-0.032	[-0.064, 0.001]
				2014 vs. 2007	-0.021	[-0.031, -0.010]
				2014 vs. 2000	-0.052	[-0.084, -0.020]

^Ɨ^ linear regression model adjusted for sex, age, age^2^, education, diabetes duration, history of CVD, (and fasting status).

^#^ linear regression model adjusted for sex, age, age^2^, education, diabetes duration, history of CVD, and fasting status restricted for participants without a prior CVD event (MI, stroke).

### Comorbidities and Quality of life

**Fig 6:**
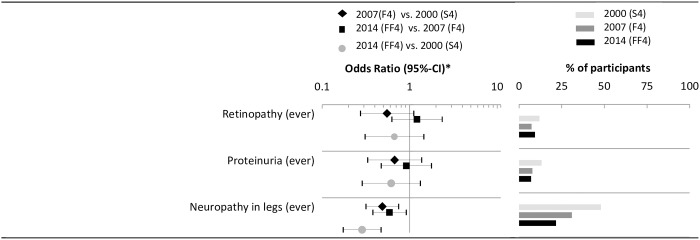
Time trends of microvascular complications. * Logistic regression model adjusted for sex, age, age^2^, education, diabetes duration, and history of CVD.

The odds of having retinopathy and proteinuria insignificantly decreased from 2000 to 2014. The odds of having leg neuropathy halved between 2000 and 2007 (OR_2007 vs. 2000_ = 0.61 [95%-CI: 0.40; 0.93]) and between 2007 and 2014 (OR_2014 vs. 2007_ = 0.44 [0.29; 0.67]) (Fig 6).

The PCS score moderately, but significantly improved from 2007 to 2014 (adjusted difference_2014 vs. 2007_ = 2.52 points [95%-CI: 0.36, 4.69]. No significant time trend was observed for the MCS score (Table 3). All prevalances and odds ratios which are graphically illustrated in Figs 2–6 are provided as numbers in S2–S4 Tables.

**Table 3:** Time trends of health-related quality of life (HRQL) Ɨ.

	S4 study	F4 study	FF4 study	Association		
	1999–2001	2006–2008	2013/2014			
	Adjusted mean	Adjusted mean	Adjusted mean	Comparison	Adjusted difference	[95%- CI]
SF-12 physical component summary	41.1	41.2	43.6	2007 vs. 2000	0.13	[-1.94, 2.20]
				2014 vs. 2007	2.39	[0.50, 4.28]
				2014 vs. 2000	2.52	[0.36, 4.69]
SF-12 mental component summary	50.6	49.5	51.1	2007 vs. 2000	-1.11	[-3.43, 1.21]
				2014 vs. 2007	1.64	[-0.48, 3.76]
				2014 vs. 2000	0.53	[-1.89, 2.95]

^Ɨ^ linear regression model adjusted for sex, age, age^2^, education, diabetes duration, and history of CVD.

### Sensitivity analyses

We compared the results of the analyses between patients with a diabetes duration of <7 years, and of patients with diabetes duration of ≥7 years. The first subsample comprises participants from FF4 and F4 whose diabetes was not already prevalent in the previous survey, i.e. ‘incident cases’ and those from S4 whose diabetes was diagnosed within the last 7 years. Although some effects tended to be stronger or weaker in some strata, no systematic pattern could be detected.

## Conclusions

High quality care, including adequate monitoring, control of risk factors and active self-management are the key factors for preventing costly and burdensome micro- and macrovascular complications in patients with type 2 diabetes. Little is known about time trends in the quality of diabetes care in Germany in recent years. The goal of this study was to describe the development of quality of diabetes care from 2000 to 2007 to 2014 using population-based data from a regional cohort study. Results were mixed: Deteriorations or no improvements were observed for the majority of indicators of patient self-management and physician-delivered care. Medication and glycemic control, blood pressure control and lipid control improved, which may translate into a considerable reduced CHD and stroke risk, however, quality of care remained still suboptimal. In parallel to these trends, the prevalence of microvascular complications decreased and HRQL improved.

The increase of glucose lowering and cardio-protective medication and the improvements in the control of cardiovascular risk factors from 2000 to 2014 was substantial. For example, the odds of having a blood pressure of < 140/80 mmHg were around 6 times higher in 2014 compared with the year 2000. This finding supports the results from studies on data from 3 regional population-based surveys of the German DIAB-CORE consortium and from data of the German National Health Interview and Examinations Surveys (GNHIES) in which both the treatment and control for blood pressure was found to have increased between 1997 and 2011 [23, 24]. Also internationally the control of blood pressure improved during this time period [30]. Our study further shows that the odds of receiving lipid lowering medication and of having LDL levels <2.6 mmol/l increased between 2000 and 2014 by the factors 3.6 and 2.6, respectively. Besides observing higher odds for reaching treatment targets we also observed a shift in the overall distributions of HbA1c, blood pressure and cholesterol. As a result of these distribution shifts the modeled absolute 10 year risk for CHD events and stroke decreased substantially.

Noteworthy, except for oral antidiabetic therapy and control of HDL cholesterol, improvements in medication and risk factor control were large comparing 2007 with 2000, but small or non-existent comparing 2014 with 2007. Similar as observed in this study, Schunk et al. and Du et al. reported in previous work that the proportion of German patients who received regular HbA1c checks and medication with cardio-protective drugs and those with well-controlled blood pressure and lipids increased substantially from 2000 to 2008 [21, 22] and 1997–99 to 2008–11 [24]. This improvement in quality of care in the 2000s years might be related to the introduction of structured DMPs (2003) for type 2 diabetes [18] as several studies indicated that the introduction of DMPs improved the quality of care of participating patients [31, 32] or even caused a spill-over effect which increased the quality of care of all patients with diabetes [33]. To date it has remained unclear if and to which magnitude this positive trend has remained. This study indicates that beyond a few exceptions the large improvements in quality of care (oral antidiabetic medication, HDL-cholesterol control) that were observed in the 2000s years attenuated in recent years.

This flattening of quality of care improvements in recent years is problematic as the current control of glucose and cardiovascular risk factors is still sub-optimal. In 2014, 29% of participants with diabetes still did not reach an HbA1c of <7%, 31% did not reach a blood pressure of <140/80 mm/Hg, and 73% did not reach a LDL cholesterol goal of <2.6 mmol/l. Particularly, improvements of lipid control occurred on a very poor level. This finding supports results from the ‘Guideline Adherence to Enhance Care (GUIDANCE)’ study in 2009/10 which compared quality of diabetes care in 8 European countries including Germany. This study showed that the general quality of care in Germany was comparable to other countries, but that the proportion of participants with diabetes on lipid lowering medication (46% in Germany vs. 68% on average) and with a LDL <2.6 mmol/l (31% in Germany vs. 55% on average) was considerably lower than in the other 7 European countries. More effort should therefore be put on convincing reluctant general practitioners and patients from the benefits of lipid-lowering medication and enforcing evidence-based treatment guidelines for lipid control [29, 34]

Beside improvements in quality of care indicators, the prevalence of self-reported microvascular complications, in particular neuropathy, decreased remarkably over the 14 year time frame. Although the validity of patient self-reports on complications is unknown, this positive trend might be the result of better risk factor control and improved standards in monitoring and screening. The finding is also in line with data from the US, England and Finland, where decreasing rates of amputations and end stage renal disease indicated a decline in microvascular complications between 2000 and 2010 [27, 35, 36]. Also, the improvement in the physical component of the SF-12 is likely to be related to the lower microvascular burden, which is known to have a strong negative impact on HRQL [37].

Though the medication for and control of blood glucose and cardiovascular risk factors improved over the last 14 years, the quality of patient self-management has remained unchanged on a rather poor level or even decreased in recent years. This is firstly alarming, as patient self-management behavior is known to be predictive for glycemic control and long-term all-cause mortality [25] and secondly unexpected, as with the initiation of German DMPs the active engagement of patients in the disease management process should have been fostered [33, 38]. Whereas in the GNHIES the proportion of participants with diabetes who regularly monitor their blood glucose increased from 38% in 1997–1999 to 64% in 2008–2011, the proportion in our study decreased from 55% in 2007 to 43% in 2014. Our data further shows that in 2014 not even half of participants with diabetes have ever participated in a patient education class (compare Fig 2/S2 Table). In additional analyses we revealed that participation in a diabetes education class was positively correlated with some dimensions of self-management behavior (e.g. self-monitoring of blood glucose) highlighting the importance of educational strategies. Large efforts are therefore needed to extend the reach of existing education programs to improve patient self-management.

The strength of our study is its population-based design, its long follow-up time, the identification of validated type 2 diabetes cases and the standardized and comprehensive assessment of quality of care, including patient self-management, physician-delivered examinations and counseling, medication and clinical outcome measures.

When interpreting the data, a few limitations should be considered. The data stem from a regional cohort study in Southern Germany and is probably not generalizable for the rest of Germany. Previous studies have shown that there are regional differences in the quality of diabetes care in Germany. It is known that the quality of care in the southern part of Germany, from where the cohort study recruits its participants, is above the national average [39]. Furthermore the (potential) selective participation is a limitation. Firstly, this effect could reduce the external validity of the study. Secondly it is possible that through the repeated self-selection process healthier and better controlled participants tended to re-participate in the follow-up studies. Another potential problem one should be aware of is that despite of the similar diabetes duration at time of examinations, the lead time between clinical onset and diagnosis of diabetes probably decreased over the last 15 years. The reduced lead time, resulting in earlier detected and therefore ‘healthier’ patients might be one source of residual confounding that we could not control for. Both, the self-selection of healthier participants and the reduced lead time are expected to cause an overestimation of quality of care improvements or an underestimation of quality of care deteriorations. This limitations needs to be considered in the interpretation of the results.

Furthermore, although all assessments and measurements were performed under standardized conditions, the laboratory methods of measuring HbA1c, blood pressure and lipids changed over the studies. Therefore, it cannot be ruled out that the magnitude of effects observed in the clinical outcome measures might be partly over- or underestimated. It also has to be acknowledged that the operationalization of patient self-management is quite difficult. The used questionnaire comprises similar items as other instruments self-management questionnaires, however, other important dimensions such as medication adherence are not included [25, 40]. Another limitation is the self-reported nature of the data. Whereas for patient self-management self-reports might be the only usable data source, the validity of information on physician-delivered care via self-reports is probably inferior compared to studies based on administrative claims data of health insurances [41]. Moreover, risk equations of the UKPDS risk engine are derived from a historic cohort and it is known that the model overestimates the absolute risk for cardiovascular events in current patients [42]. Despite this limitation, we decided to report modeled cardiovascular risk, as we think the benefit of having a summarized unidimensional risk measure that combines information from behavioral and clinical risk factors outweighs potential inaccuracies in the absolute risk.

## Conclusions

Medication and risk factor control improved from 2000 to 2007 to 2014 with a flattening of improvements from 2007 to 2014, resulting in currently still suboptimal care. No improvements or even deteriorations over the 14 years were observed in the level of patient self-management. Continuous effort, including the enforcement of evidence-based treatment and active patient-self management education is needed to further improve quality of care and to reduce the patients’ risk for developing costly and burdensome micro- and macrovascular complications.

Due to selection or lead time bias an overestimation of quality of care improvements cannot be ruled out. Repeated cross-sectional surveys with standardized instruments are needed for further clarification of the trends in quality of type 2 diabetes care in Germany.

## Supporting Information

S1 Table Overview on Laboratory Measurement Methods. ^†^ After about half of the study period, the KORA FF4 measurement instrument and assays changed from Siemens to Roche. Calibration formulas were developed using 122 KORA FF4 samples which were measured with both instruments / assays during the time of the method change. The Siemens measurement results were calibrated to correspond to the Roche measurements using the following formulas [all units in mg/dl]: Total_Cholesterol_Roche = 3.00 + Total_Cholesterol_Siemens * 1.00; HDL_Cholesterol_Roche = 2.40 + HDL_Cholesterol_Siemens * 1.12; LDL_Cholesterol_Roche = antilog (-0.13328 + log LDL_Cholesterol_Siemens * 1.03051); Triglycerides_Roche = 4.97073 + Triglycerides_Siemens * 0.90732.
(PDF)

S2 Table Time trends of patient self-management Ɨ. Ɨ logistic regression model adjusted for sex, age, age^2^, education, diabetes duration, and history of CVD. (PDF)

S3 Table Time trends of physician-delivered care Ɨ. Ɨ logistic regression model adjusted for sex, age, age^2^, education, diabetes duration, and history of CVD. (PDF)

S4 Table Time trends of medication use, of reaching treatment targets and of microvascular complications. ⊣ based on guidelines of the German Diabetes Association (DDG) 2014. ⌉ based on guidelines of the American Diabetes Association (ADA) 2014. Ɨ logistic regression model adjusted for sex, age, age^2^, education, diabetes duration, history of CVD, (and fasting status).
(PDF)
