# Supplementary material for: Quality of Diabetes Care in Germany Improved from 2000 to 2007 to 2014, but Improvements Diminished since 2007. Evidence from the Population-Based KORA Studies
Source: PLoS One. 2016 Oct 17;11(10):e0164704. doi: 10.1371/journal.pone.0164704 (PMC5066975; doi:10.1371/journal.pone.0164704)
Supplement: S1 Table — † After about half of the study period, the KORA FF4 measurement instrument and assays changed from Siemens to Roche. Calibration formulas were developed using 122 KORA FF4 samples which were measured with both instruments / assays during the time of the method change. The Siemens measurement results were calibrated to correspond to the Roche measurements using the following formulas [all units in mg/dl]: Total_Cholesterol_Roche = 3.00 + Total_Cholesterol_Siemens * 1.00; HDL_Cholesterol_Roche = 2.40 + HDL_Cholesterol_Siemens * 1.12; LDL_Cholesterol_Roche = antilog (-0.13328 + log LDL_Cholesterol_Siemens * 1.03051); Triglycerides_Roche = 4.97073 + Triglycerides_Siemens * 0.90732. (PDF) [file pone.0164704.s001.pdf]

S1 Table. Overview on Laboratory Measurement Methods.

**Outcome**	**S4 Study (1999-2001)**	**F4 Study (2006-2008)**	**FF4 Study (2013-2014)**
**Blood Pressure**	Automatic device<br>Type: HEM-705CP<br>(Fa. OMRON HEALTHCARE GmbH)	Digital device<br>Type: HEM-705CP (Fa. OMRON HEALTHCARE GmbH)	Digital device<br>Type: HEM-705CP<br>(Fa. OMRON HEALTHCARE GmbH)
**Total Cholesterol (serum)**	Enzymatic, photometric CHOL assay on a Hitachi 717 instrument (Roche Diagnostics GmbH, Mannheim, Germany).	Enzymatic, colorimetric CHOL Flex assay on a Dimension RxL instrument (Dade Behring Inc., Newark, USA).	Enzymatic, colorimetric CHOL Flex assay on a Dimension Vista 1500 instrument (Siemens Healthcare Diagnostics Inc., Newark, USA) or enzymatic, colorimetric CHOL2 assay on a Cobas c701/702 instrument (Roche Diagnostics GmbH, Mannheim, Germany).<br>For details please refer to footnote †
**LDL Cholesterol (serum)**	Determination of non-LDL Cholesterol by use of the enzymatic, photometric CHOL assay on a Hitachi 717 instrument (Roche Diagnostics GmbH, Mannheim, Germany) after precipitation of LDL Cholesterol by QUANTOLIP reagents (Immuno AG, Vienna, Austria). LDL Cholesterol is calculated as the difference between Total and non-LDL Cholesterol.	Enzymatic, colorimetric ALDL Flex assay on a Dimension RxL instrument (Dade Behring Inc., Newark, USA).	Enzymatic, colorimetric LDLC Flex assay on a Dimension Vista 1500 instrument (Siemens Healthcare Diagnostics Inc., Newark, USA) or enzymatic, colorimetric LDL_C assay on a Cobas c701/702 instrument (Roche Diagnostics GmbH, Mannheim, Germany).<br>For details please refer to footnote †
**HDL Cholesterol (serum)**	Enzymatic, photometric CHOL assay on a Hitachi 717 instrument after precipitation of non-HDL Cholesterol by HDL-C reagents (Roche Diagnostics GmbH, Mannheim, Germany).	Enzymatic, colorimetric ALDL Flex assay on a Dimension RxL instrument (Dade Behring Inc., Newark, USA).	Enzymatic, colorimetric HDLC Flex assay on a Dimension Vista 1500 instrument (Siemens Healthcare Diagnostics Inc., Newark, USA) or enzymatic, colorimetric HDLC3 assay on a Cobas c701/702 instrument (Roche Diagnostics GmbH, Mannheim, Germany).<br>For details please refer to footnote †
**Triglycerides (serum)**	Enzymatic, colorimetric TG assay on a Hitachi 917 instrument (Roche Diagnostics GmbH, Mannheim, Germany).	Enzymatic, colorimetric TGL Flex assay on a Dimension RxL instrument (Dade Behring Inc., Newark, USA).	Enzymatic, colorimetric TRIG Flex assay on a Dimension Vista 1500 instrument (Siemens Healthcare Diagnostics Inc., Newark, USA) or enzymatic, colorimetric TRIGL assay on a Cobas c701/702 instrument (Roche Diagnostics GmbH, Mannheim, Germany).<br>For details please refer to footnote †
HbA1c (hemolyzed whole blood)	Turbidimetric inhibition immunoassay Tina-Quant HBA1C II on a Hitachi 717 instrument (Roche Diagnostics GmbH, Mannheim, Germany)	Cation-exchange high performance liquid chromatographic, photometric assay on an Adams HA 8160 Hemoglobin Analysis System (Arkray Inc., distributed by A. Menarini Diagnostics, Florence, Italy)	Cation-exchange high performance liquid chromatographic, photometric VARIANT II TURBO HbA1c Kit - 2.0 assay on a VARIANT II TURBO Hemoglobin Testing System (Bio-Rad Laboratories Inc., Hercules, USA)

† After about half of the study period, the KORA FF4 measurement instrument and assays changed from Siemens to Roche. Calibration formulas were developed using 122 KORA FF4 samples which were measured with both instruments / assays during the time of the method change. The Siemens measurement results were calibrated to correspond to the Roche measurements using the following formulas [all units in mg/dl]: Total_Cholesterol_Roche = 3.00 + Total_Cholesterol_Siemens * 1.00; HDL_Cholesterol_Roche = 2.40 + HDL_Cholesterol_Siemens * 1.12; LDL_Cholesterol_Roche = antilog (-0.13328 + log LDL_Cholesterol_Siemens * 1.03051); Triglycerides_Roche = 4.97073 + Triglycerides_Siemens * 0.90732.
