# Supplementary material for: Quality of Diabetes Care in Germany Improved from 2000 to 2007 to 2014, but Improvements Diminished since 2007. Evidence from the Population-Based KORA Studies
Source: PLoS One. 2016 Oct 17;11(10):e0164704. doi: 10.1371/journal.pone.0164704 (PMC5066975; doi:10.1371/journal.pone.0164704)
Supplement: S2 Table — (PDF) [file pone.0164704.s002.pdf]

S2 Table. Time trends of patient self-management Ɨ.

	S4 study	F4 study	FF4 study	Association		
	1999-2001	2006-2008	2013/2014			
	%	%	%	Comparison	**Odds Ratio Ɨ**	[95%- CI]
Self-monitoring of blood glucose (≥1 times/week)	No data	55.3	42.6	F4 vs. S4		
				FF4 vs. F4	0.56	[0.39, 0.81]
				FF4 vs. S4		
Self-monitoring of blood pressure (≥1 times/week)	55.0	53.6	48.5	F4 vs. S4	0.86	[0.56, 1.31]
				FF4 vs. F4	0.82	[0.59, 1.15]
				FF4 vs. S4	0.70	[0.43, 1.15]
Self-monitoring of body weight (≥1 times/week)	No data	53.8	55.3	F4 vs. S4		
				FF4 vs. F4	1.11	[0.77, 1.61]
				FF4 vs. S4		
Checking feet (≥1 times/week)	No data	56.6	50.2	F4 vs. S4		
				FF4 vs. F4	0.80	[0.56, 1.16]
				FF4 vs. S4		
Not smoking (currently)	86.0	89.2	91.5	F4 vs. S4	1.19	[0.75, 1.88]
				FF4 vs. F4	1.01	[0.65, 1.56]
				FF4 vs. S4	1.20	[0.65, 2.22]
Physical Activity (≥2hours/week)	13.3	12.3	16.5	F4 vs. S4	1.01	[0.53, 1.93]
				FF4 vs. F4	1.20	[0.71, 2.03]
				FF4 vs. S4	1.22	[0.62, 2.38]
Participation in a diabetes education class (ever)	47.3	54.0	43.1	F4 vs. S4	1.62	[1.07, 2.44]
				FF4 vs. F4	0.63	[0.44, 0.90]
				FF4 vs. S4	1.01	[0.62, 1.64]
Having a diabetes diary (currently)	71.0	58.0	40.0	F4 vs. S4	0.41	[0.26, 0.64]
				FF4 vs. F4	0.60	[0.38, 0.96]
				FF4 vs. S4	0.25	[0.14, 0.42]

Ɨ logistic regression models adjusted for sex, age, age^2, education, diabetes duration, and history of CVD.
