# Supplementary material for: Quality of Diabetes Care in Germany Improved from 2000 to 2007 to 2014, but Improvements Diminished since 2007. Evidence from the Population-Based KORA Studies
Source: PLoS One. 2016 Oct 17;11(10):e0164704. doi: 10.1371/journal.pone.0164704 (PMC5066975; doi:10.1371/journal.pone.0164704)
Supplement: S3 Table — (PDF) [file pone.0164704.s003.pdf]

S3 Table. Time trends of physician-delivered care Ɨ.

	S4 study	F4 study	FF4 study	Association		
	1999-2001	2006-2008	2013/2014			
	%	%	%	Comparison	**Odds Ratio**	[95%- CI]
Control of HbA1c (last 12 months)	29.3	52.5	71.6	F4 vs. S4	3.22	[2.07, 5.02]
				FF4 vs. F4	2.55	[1.70, 3.82]
				FF4 vs. S4	8.20	[4.83, 13.94]
Control of blood pressure (last 12 months)	No data	95.5	96.1	F4 vs. S4		
				FF4 vs. F4	1.21	[0.50, 2.92]
				FF4 vs. S4		
Control of cholesterol (last 12 months)	No data	90.6	87.8	F4 vs. S4		
				FF4 vs. F4	0.77	[0.42, 1.42]
				FF4 vs. S4		
Control of protein in urine (last 12 months)	No data	73.1	61.7	F4 vs. S4		
				FF4 vs. F4	0.59	[0.39, 0.90]
				FF4 vs. S4		
Eye examination (last 12 months)	61.1	72.6	63.8	F4 vs. S4	2.77	[1.75, 4.37]
				FF4 vs. F4	0.79	[0.55, 1.15]
				FF4 vs. S4	2.19	[1.35, 3.57]
Foot examination (last 12 months)	36.9	57.0	51.7	F4 vs. S4	1.61	[1.05, 2.48]
				FF4 vs. F4	0.63	[0.42, 0.94]
				FF4 vs. S4	1.01	[0.63, 1.61]
Counselling on topic 'physical activity' (last 12 months)	No data	56.0	48.1	F4 vs. S4		
				FF4 vs. F4	0.70	[0.47, 1.05]
				FF4 vs. S4		
Counselling on topic 'diet' (last 12 months)	No data	58.3	44.4	F4 vs. S5		
				FF4 vs. F5	0.59	[0.40, 0.86]
				FF4 vs. S5		

Ɨ logistic regression models adjusted for sex, age, age^2 education, diabetes duration, and history of CVD
