# Supplementary material for: Quality of Diabetes Care in Germany Improved from 2000 to 2007 to 2014, but Improvements Diminished since 2007. Evidence from the Population-Based KORA Studies
Source: PLoS One. 2016 Oct 17;11(10):e0164704. doi: 10.1371/journal.pone.0164704 (PMC5066975; doi:10.1371/journal.pone.0164704)
Supplement: S4 Table — ⊣ based on guidelines of the German Diabetes Association (DDG) 2014. ⌉ based on guidelines of the American Diabetes Association (ADA) 2014. Ɨ logistic regression model adjusted for sex, age, age2, education, diabetes duration, history of CVD, (and fasting status). (PDF) [file pone.0164704.s004.pdf]

S4 Table. Time trends of medication use, of reaching treatment targets and of microvascular complications Ɨ.

	S4 study	F4 study	FF4 study	Association		
	1999-2001	2006-2008	2013/2014			
	%	%	%	Comparison	**Odds Ratio**	[95%- CI]
**Medication Use**						
Intake of oral glucose lowering medication (last 7 days)	70.7	71.9	84.4	F4 vs. S4	1.07	[0.67, 1.70]
				FF4 vs. F4	2.34	[1.48, 3.68]
				FF4 vs. S4	2.50	[1.44, 4.34]
Intake of insulin (last 7 days)	22.0	23.2	13.3	F4 vs. S4	1.20	[0.67, 2.14]
				FF4 vs. F4	0.39	[0.24, 0.65]
				FF4 vs. S4	0.47	[0.22, 0.98]
Intake of blood pressure lowering medication (last 7 days)	62.0	76.4	75.4	F4 vs. S4	1.77	[1.14, 2.74]
				FF4 vs. F4	0.73	[0.49, 1.10]
				FF4 vs. S4	1.30	[0.80, 2.09]
Intake of lipid lowering medication (last 7 days)	18.7	40.4	45.5	F4 vs. S4	2.96	[1.80, 4.86]
				FF4 vs. F4	1.18	[0.83, 1.68]
				FF4 vs. S4	3.50	[2.02, 6.06]
Intake of platelet inhibiting medication (last 7 days)	31.3	34.0	32.7	F4 vs. S5	0.89	[0.56, 1.43]
				FF4 vs. F5	0.79	[0.52, 1.23]
				FF4 vs. S5	0.71	[0.42, 1.21]
**Reaching treatment targets**						
HbA1c < 7,0% ˧	60.5	66.7	71.3	F4 vs. S4	1.20	[0.77, 1.86]
				FF4 vs. F4	1.30	[0.85, 1.98]
				FF4 vs. S4	1.56	[0.93, 2.60]
Bloodpressure < 140 mmHg (syst.) and < 80 mmHg (diast.) ˧	25.3	63.6	68.9	F4 vs. S4	5.25	[3.34, 8.25]
				FF4 vs. F4	1.17	[0.78, 1.76]
				FF4 vs. S4	6.14	[3.73, 10.09]
BMI < 30kg/m2	41.2	43.9	43.9	F4 vs. S4	0.87	[0.64, 1.18]
				FF4 vs. F4	0.85	[0.63, 1.14]
				FF4 vs. S4	0.74	[0.48, 1.13]
LDL-cholesterol < 2.6 mmol/l ˧	12.9	22.8	27.4	F4 vs. S4	4.56	[2.17, 9.54]
				FF4 vs. F4	1.20	[0.77, 1.87]
				FF4 vs. S4	5.47	[2.52, 11.84]
HDL cholesterol >1.0 mmol/l in men and >1.3 mmol/l in women ˥	51.0	59.4	75.0	F4 vs. S4	1.18	[0.49, 2.82]
				FF4 vs. F4	2.69	[1.83, 3.94]
				FF4 vs. S4	3.17	[1.24, 8.13]
Triglycerids <1.71 mmol/l ˥	23.2	52.5	60.4	F4 vs. S4	1.06	[0.48, 2.35]
				FF4 vs. F4	1.23	[0.88, 1.72]
				FF4 vs. S4	1.31	[0.57, 3.04]
**Microvascular complications**						
Retinopathy (ever)	12.0	7.4	9.4	F4 vs. S4	0.55	[0.27, 1.11]
				FF4 vs. F4	1.21	[0.62, 2.36]
				FF4 vs. S4	0.67	[0.31, 1.45]
Proteinuria (ever)	13.3	7.9	7.1	F4 vs. S4	0.73	[0.36; 1.45]
				FF4 vs. F4	0.84	[0.44; 1.61]
				FF4 vs. S4	0.61	[0.28; 1.32]
Neuropathy in legs (ever)	48.0	31.0	21.7	F4 vs. S4	0.44	[0.29; 0.67]
				FF4 vs. F4	0.61	[0.40; 0.93]
				FF4 vs. S4	0.27	[0.16; 0.44]

˧ based on guidelines of the German Diabetes Association (DDG) 2014. ˥ based on guidelines of the American Diabetes Association (ADA) 2014. Ɨ logistic regression model adjusted for sex, age, age^2, education, diabetes duration, history of CVD, (and fasting status).
